# Reliability and validity of simplified Chinese version of the Italian spine youth quality of life questionnaire in adolescents with idiopathic scoliosis

**DOI:** 10.1186/s12891-021-04462-8

**Published:** 2021-06-21

**Authors:** Shanshan Liu, Junyang Liang, Nanfang Xu, Shuo Mai, Qi Wang, Lin Zeng, Chaojun Du, Yaoxu Du, Yan Zeng, Miao Yu, Zhongjun Liu

**Affiliations:** 1 Department of Orthopaedic Surgery, Peking University Third Hospital, 100191 Beijing, China; 2 Department of Spinal Surgery, Weihai Wei People’s Hospital, Weihai, China; 3 Peking University Health Science Center, Beijing, China; 4 Research Center of Clinical Epidemiology, Peking University Third Hospital, Beijing, China; 5 Department of Orthotics and Prosthetics, Peking University Third Hospital, Beijing, China

**Keywords:** Quality of life, Adolescent idiopathic scoliosis, Cultural adaptation, Italian Spine Youth Quality of Life

## Abstract

**Background:**

The Italian Spine Youth Quality of Life (ISYQOL) questionnaire is used to evaluate health-related quality of life in adolescents with Idiopathic Scoliosis. The study aimed to undertake the process of cultural adaptation of the ISYQOL questionnaire into Simplified Chinese.

**Methods:**

Translate from Italian into Simplified Chinese. It involved 138 adolescents whose Cobb angle ranged between 20 and 40 degrees, 50 wearing the brace and 88 not wearing the brace. Statistical analysis calculated the reliability, floor effects, and ceiling effects of the ISYQOL. After that, construct validity was measured by analyzing the ISYQOL relationship Scoliosis Research Society-22 patient Questionnaire (SRS-22).

**Results:**

There were no floor or ceiling effects in the ISYQOL questionnaire. Cronbach’s alpha coefficient evaluated for Internal consistency was 0.75 in the no-treated group and 0.88 in the brace-treated group. Intraclass correlation coefficients assessed with the use of the test-retest method were 0.72 in the no-treated group and 0.80 in the brace-treated group. A strong relationship exists between the ISYQOL measure and SRS-22 scores (rho = 0.62; *p* < 0.01), reflecting the high validity of the questionnaires. Both ISYQOL and SRS-22 scores showed no statistical difference between groups wearing and not wearing the brace (*p* > 0.05).

**Conclusions:**

Trans-cultural validation in Chinses language showed the reliability and validity of the ISYQOL.

## Background

Adolescent Idiopathic Scoliosis (AIS) is a frequent pathology among adolescents, the prevalence of which is 1–2 % [1]. Scoliosis itself and its treatment, including bracing and surgery, may have a severe impact on the patient’s health-related quality of life (HRQOL) [2]. The need for a specific HRQOL measure in AIS has been recognized for a long time, which seems as essential as radiologic measurements in evaluating treatment effect [3]. As a way of measuring HRQOL, self-administered questionnaires have become the most commonly used means to assess the patients’ perspective of health. In 2017 Caronni et al. [4] proposed the ISYQOL questionnaire, which showed high measurement properties in Rasch analysis. Previously, several questionnaires such as Scoliosis Research Society-24 (SRS-24) and Scoliosis Research Society-22 (SRS-22) have been developed to evaluate HRQOL in spinal deformities, but have poor measurement properties. In previous work, the Rasch analysis has used to test SRS-22, a refinement of the SRS-24, in the item response theory (IRT) framework showing disordered thresholds, overall misfit, and ceiling effect [4, 5]. So ISYQOL questionnaire was developed as a new HRQOL measure. The original version was in Italian. The English version of the ISYQOL is simply the forward translation of the Italian one, and the subsequent stages of the forward- and back-translation process remain to be developed. Several research groups worldwide are already working on a proper translation of ISYQOL. Further research has found this questionnaire of adolescents with spinal deformities better than the SRS-22 questionnaire. ISYQOL showed high validity when used to measure HRQOL in adolescents with spinal deformities. Moreover, ISYQOL performs better than SRS22, having better known-groups validity and (contrary to SRS22) detecting the impact of disease severity on HRQOL [6], which seems to be the most widely used as a reference standard in the past [7]. ISYQOL was developed and validated in the Italian language. To be used in a different language-speaking population, it has to be subject to a process called trans-cultural validation [8]. The reliability and validity of self-administered questionnaires need to evaluate to make them suitable for research and clinical practice [9]. Simplified Chinese is the contemporary Chinese primarily used in mainland China, Singapore, and Malaysia. The study aimed to evaluate the reliability and validity of the Simplified Chinese version of the ISYQOL (SC-ISYQOL) in adolescents with idiopathic scoliosis.

## Methods

### Questionnaire

ISYQOL Questionnaire is a 20 Likert scale questionnaire and consisted of two domains (13 items of the spine health and 7 of the brace domains). Items in the spine health domain are about the condition of scoliosis, while items in the brace domain are about the impact of wearing a brace. The first version was designed to be administered to patients wearing the brace and consisted of both the spine health and brace domains (20 items). The second version designed to be distributed to patients not wearing the brace and consisted of the spine health domain only (13 items). Scoring of the ISYQOL planned as follows: Items investigating the presence of spine-related problems coded 0, 1, or 2 (0, never; 1, sometimes; 2, often). Items investigating the presence of positive thoughts coded 2, 1, or 0 (2, never; 1, sometimes; 0, often). The ISYQOL total score was obtained by adding up all single items, 40 in the brace-treated version, and 26 in the no-treated version. The ordinal ISYQOL total score could be converted to an interval measure (i.e., ISYQOL measure), which is expressed on a 0–100 % scale (with 100 % indicating the excellent quality of life). ISYQOL measure was a percentage making patients not wearing or wearing the brace comparable who filled in different versions of the questionnaire. SRS-22 comprises 5 domains (function, pain, self-image, mental health, and satisfaction/dissatisfaction) up to a total of 22 items. The score ranges from 1 to 5 points for each item, with a summary score between 22 and 110 [7]. Higher scores indicated better quality of life. In our study, we used Simplified Chinese validated version of SRS-22 (SC-SRS-22) [10, 11].

### Translation

We followed widely accepted guidelines described by Guillemin for translation and cross-cultural adaptation of ISYQOL questionnaires [7, 8]. In the first stage, two independent translators converted the original Italian text into Simplified Chinese, whose native language is Italian. One of the translators, who had a medical background, was instructed on the whole process of adaptation. The other translator had no medical background and received no information on the project. The second stage consisted of a comparison of the original and two translated versions. During that stage, the two translators and the authors identified differences in translations and produced a combined version. In the third stage—the so-called reversed translation—two independent translators, who were native in Italy, translated the Simplified Chinese version into the language of the original document (Italian). The translators were not familiar with the original version. The objective of this stage was to assure the equivalence of the two versions and identify possible mistranslations. At the last fourth stage, a commission composed of a specialist in orthopedics, translators, a statistician, and a psychologist reviewed the translations without commitment to modify. As a result of consensus, the final version of the SC-ISYQOL was drafted.

### Sample

The study took place from February 2019 to November 2019 with the approval of the local Hospital Ethics Committee. The patients’ sample split as follows: 1. with idiopathic scoliosis; 2. aged 10–18 years; 3. Cobb (the angle of curvature of the spine) 20–40 degrees; 4 without a history of spine surgery; 5.no-treated or brace-treated more than 3 months. At the end of the clinical visit, a dedicated research assistant administered the SC-ISYQOL questionnaire, which is the same version as the “final version SC-ISYQOL” mentioned above, and the SC-SRS-22 questionnaire to patients separately after obtaining patients and their guardians‘ consent. The research assistant was responsible for ascertaining the on-site completion of all questionnaire items by the participants during their office visits. Then they collected the questionnaire and entered it into the database. The study included 138 consecutive patients. To reduce the cost of the experiment, we invite part of the patients passing a random number to interview again after two weeks and fill out the questionnaire again. So that 70 patients had the test-retest using the same method. We set two weeks as the interval between the assessments based on previous research [12], which also mentioned the clinical status is unlikely to change appreciably in the absence of intervention during this time.

### Statistical analysis

Statistical analysis performed using Statistics 26.0 software. Descriptive statistics used to calculate mean scores and standard deviations for a given question and a domain. The independent-sample t test used to compare the differences in QOL scores between the subgroups based on brace using. The two most important properties of reliability are consistency and stability. Internal consistency assessed using Cronbach’s alpha coefficient. A test-retest design was used to measure the temporal stability of the questionnaire with intraclass correlation coefficient (ICC). Construct validity was assessed by comparing the ISYQOL measure with SRS-22 through Spearman rank correlation coefficient (r) in total and the subgroups based on gender, age, and brace using.

For all tests, statistical significance was set at *p* < 0.05.

## Results

The sample included 113 girls and 25 boys, whose mean age at the time of questionnaire administration was 13.7 years (± 2.3 years). The Cobb angle ranged between 21.1 and 38.5 degrees, and for demographic and clinical characteristics details, see Table 1.

**Table 1:** Participants’ demographics and clinical data

N	138
Males vs. females, N	25 vs. 113
Mean age (SD), years	13.7 (2.3)
Mean body weight (SD), kg	47.1 (11.7)
Mean height (SD), cm	160.4 (12.2)
Cobb angle, Min-Max	21.1–38.5
Lenke type, MT vs. DM vs. TL/L	41 vs. 58 vs. 39
No brace vs. brace	88 vs. 50
Duration of bracing, Min-Max, months	4–45
Daily wearing time (SD), hours	16.3 (5.9)
Test-retest, no brace vs. brace N	44 vs. 26

*N* number of participants, *SD* standard deviation, *Min *minimal, *Max* maximal

There are only 16 (0.6 %) missing items in all patients’ questionnaires, and we use mean to fill in missing values in this study. A dedicated research assistant ensures better questionnaire quality. The mean and standard deviation of ISYQOL and SRS-22 scores are presented in Table 2. Patients not wearing the brace only filled out the spine health domain. The mean score for ISYQOL was 9.4 (ranged between 1 and 20) in the no-treated group, while 14.2 (ranged between 2 and 33) in the brace-treated group. Both ISYQOL and SRS-22 scores showed no statistical difference between subgroups wearing and not wearing the brace (p > 0.05). There are no floor and ceiling effects in the ISYQOL questionnaire in both versions.

**Table 2:** Mean and standard deviation of ISYQOL and SRS-22 scores

	No brace	Brace	*P* value
Total ISYQOL scores	9.4 (4.2)	14.2 (7.4)	/
Spine health domain	9.4 (4.2)	9.0 (5.1)	0.636
Brace domain	/	5.2 (3.5)	/
ISYQOL measure (%)	56.2(11.0)	57.7(11.1)	0.507
SRS-22 scores	91.6(8.5)	89.6(10.5)	0.229

Mean (SD)

The value of Cronbach’s alpha is presented in Table 3. In the no-treated group, Cronbach’s α coefficient was 0.75 in the spine health domain. In the brace-treated group, Cronbach’s α coefficient was 0.88, 0.85 in the spine health domain and 0.86 in the brace domain. All of them indicated good internal consistency reliability.

**Table 3:** The value of Cronbach’s alpha and Intraclass correlation coefficients (ICC)

Questionnaire	Cronbach’s alpha	ICC
ISY-No brace	0.75	0.72
ISY-Brace	0.88	0.80
spine health domain	0.85	/
brace domain	0.86	/
SRS-22	0.83	0.76

Seventy (50.7 %) patients had the test-retested, including 61 girls and 9 boys, whose mean age was 13.7 years (± 2.1 years). There is no difference in demographic characteristics between these patients and overall. ICC assessed was 0.72 in the no-treated group and 0.80 in the brace-treated group. Both versions indicated good temporal stability [13].

ISYQOL measure (with 100 % indicating the excellent quality of life) converted by ISYQOL total score is possible to compare patients not wearing the brace (who fill 13 out of 20 items only) with wearing the brace (who fill the full questionnaire). The mean and standard deviation for the ISYQOL measure and SRS22 scores are presented in Table 4. The Spearman rho between ISYQOL measure and SRS-22 scores was 0.62 with a highly significant value (*p* < 0.01). The Spearman rho of subgroups were all with highly significative value, and see details in Table 4.

**Table 4:** The Spearman rho between ISYQOL measure and SRS-22 scores

	ISYQOL measure (%)	SRS-22 scores	Spearman rho
Total	56.7 (11.0)	90.9 (9.3)	0.62^**^
Males	57.3 (10.5)	88.8 (11.5)	0.72^**^
Females	56.6 (11.1)	91.3 (8.7)	0.62^**^
Age < 13 years	57.2 (12.7)	89.8 (12.4)	0.70^**^
Age 13–18 years	56.6 (10.4)	91.3 (7.9)	0.59^**^
No brace	56.2(11.0)	91.6(8.5)	0.64^**^
Brace	57.7(11.1)	89.6(10.5)	0.59^**^

Mean (SD)

^**^p < 0.01

## Discussion

HRQOL in patients with a spine deformity decreased for several reasons, especially in adolescents [14, 15]. For example, AIS can lead to physical, psychological, and social impairments that eventually impact HRQOL [14]. Besides, conservative treatment of scoliosis with a rigid brace can be harmful to affect their QOL [16].

In this study, patients in the brace-treated group took X-rays both with and without the brace. We measured Cobb angle from X-rays without the brace, and strictly chose patients with Cobb angle ranging between 20 and 40 degrees, making the sample included in the study fitted the indications for braces for AIS. When evaluating the effect of brace treatment, the changes in the radiologic measurements and in the QOL must be considered [16]. In turn, QOL is closely related to patients’ compliance with brace treatment and depends on the conservative treatment effect [17, 18].

ISYQOL questionnaire designed to evaluate the HRQOL of adolescents with idiopathic scoliosis. Some papers perceived the need to use a disease-specific questionnaire Instead of a generic questionnaire [19–21]. From the total questionnaire score, 0 % of patients scored at the floor and 0 % scored at the ceiling, showing no floor and ceiling effects.

Cronbach’s alpha is the most commonly applied statistical parameter for showing the internal consistency of an instrument [22]. The SC-ISYQOL had a high value of Cronbach’s alpha coefficient (0.88 and 0.75), both the brace-treated and the no-treated groups, exceeding the minimum recommended value of 0.70 and indicating satisfactory internal consistency as a factor of acceptable reliability of the SC-ISYQOL.

Intraclass correlation coefficients(ICC)of the SC-ISYQOL assessed using of the test–retest method was 0.72 in the no-treated group and 0.80 in the brace-treated group, showing good temporal stability.

There are some questions designed for wearing the brace in the ISYQOL questionnaire. ISYQOL measure can convert scores to standard measurement, which is used to compare the total score of the no-treated and the brace-treated groups. It is suitable for evaluating the changes of HRQOL before and after brace treatment.

Criterion validity is the correlation of a scale with a valid, accepted universally acknowledged measure of the trait or disorder under study. The outcome measures for construct validity adopted the SRS-22, most widely used as a reference standard in the past, to evaluate the relationship with another patient-oriented questionnaire not focused on brace therapy. The results of the current study showed that a strong relationship existed between the ISYQOL measure and SRS-22 scores (rho = 0.62; *p* < 0.01), reflecting the high validity of the questionnaires. And get the same strong relationship in every subgroup (*p* < 0.01). This relationship was also found in the study by Caronni al. [6], with rho = 0.71 and *p* < 0 0.01.

There is no difference in HRQOL between the brace-treated group and the no-treated group, whether through the ISYQOL or the SRS-22. Earlier reports reported that braces affecting HRQOL may occur due to factors such as stiffness of brace [16], but it did not appear in this study. That may be related to the new-design brace applied in the hospital. The brace is light and custom-made by 3D printing, having good fit and comfort. No difference may also due to part-time brave wearing (16 h per day on average). Although the impact of the brace on HRQOL is dose-independent, other influencing factors (such as avoiding school wearing) mixed, so the conclusion cannot be drawn. Caution in the interpretation is needed. That is a cross-sectional study with a different sample of the 2 groups. This view needs to be followed up and compared by the same person’s QOL score before and after brace treatment. Perhaps the daily wear time is long enough, and the patient’s adaptation and acceptance of the brace will increase. It also needs to be confirmed by further research. There was also no statistical difference in gender and age, which may be related to the consistent disease situation.

The advantage to using the ISYQOL instead of SRS-22 or other questionnaires is that it included specific questions targeting patients who were using the brace [6]. In that way, the questionnaire is more targeted and individual. Thanks to the Rasch analysis, it is possible to compare the ISYQOL measure with other questionnaire scores even if they are different [4]. ISYQOL measure also used to compare the HRQOL of patients before and after using the brace, which is not in other questionnaires targeted to patients wearing the brace [4].

The weakness of ISYQOL is that it is not as widespread as the former questionnaires. There are limited versions in different languages and has not confirmed in a wide range of population [6].

In our study, there are limitations, such as the single clinical center available in the cross-cultural adaptation process. Further test will evaluate the reliability, construct validity, and responsiveness in the Rasch framework. The ISYQOL questionnaire needs to be promoted and applied in clinical and research, and multi-center cooperation has been obtained for further confirmation.

## Conclusions

The culturally adapted Chinese version of the ISYQOL showed good reliability, high internal consistency, and satisfactory concurrent validity. This instrument is therefore useful as a clinical evaluation tool for Chinese scoliosis patients.

## Data Availability

Data can be shared upon contact with the correspondence author.

## Abbreviations

ISYQOL: Italian Spine Youth Quality of Life
SRS-22: Scoliosis Research Society-22 patient Questionnaire
AIS: Adolescent Idiopathic Scoliosis
HRQOL: Health-related quality of life
SC-ISYQOL: Simplified Chinese version of the ISYQOL
SC-SRS-22: Simplified Chinese validated version of SRS-22
ICC: Intraclass correlation coefficient
